# Cytomegalovirus infection and rehospitalization rates after allogeneic hematopoietic stem cell and solid organ transplantation: a retrospective cohort study using German claims data

**DOI:** 10.1007/s15010-022-01847-2

**Published:** 2022-05-28

**Authors:** Daniel Teschner, Jana Knop, Christian Piehl, Sophia Junker, Oliver Witzke

**Affiliations:** 1 Department of Internal Medicine II, University Hospital Würzburg, Würzburg, Germany; 2 Department of Hematology, Medical Oncology, and Pneumology, University Medical Centre of the Johannes Gutenberg University Mainz, Mainz, Germany; 3 Takeda Pharma Vertrieb GmbH & Co. KG, Berlin, Germany; 4 Ingress-Health HWM GmbH, A Wholly Owned Subsidiary of Cytel Inc., Berlin, Germany; 5 Department of Infectious Diseases, West German Centre of Infectious Diseases, University Hospital Essen, Essen, Germany; 6 University Duisburg-Essen, Duisburg, Germany

**Keywords:** Cytomegalovirus, Allogeneic hematopoietic stem cell transplantation, Solid organ transplantation, Claims data

## Abstract

**Purpose:**

This study aimed to describe the cytomegalovirus (CMV) infection rate, rehospitalizations, and comorbidities following allogeneic hematopoietic stem cell transplantation (allo-HSCT) and solid organ transplantation (SOT).

**Methods:**

Patients who received allo-HSCT or SOT in 01/07/2015–30/06/2018 were identified using anonymized German claims data. The transplantation-related hospital admission date was defined as the index date, and patients were followed for up to 12 months (or death, first event relevant). The frequency of CMV infections (confirmed outpatient/inpatient diagnoses, ICD-10-GM codes: B25.-/B27.1) and the rate, number, and duration of all-cause rehospitalizations in the follow-up period were evaluated.

**Results:**

A total of 226 allo-HSCT and 250 SOT patients were identified (mean age 52.8 years, 38.9% female). During the 12 months after transplantation, 29.2% of allo-HSCT patients and 16.8% of SOT patients received a CMV diagnosis. The majority of these diagnoses were given during the initial hospitalization or within the following 3 months. Across transplantation types, CMV patients had more hospital readmission days per patient-year (allo-HSCT 93.3 vs. 49.4, *p* = 0.001; SOT 42.0 vs. 20.7, *p* = 0.005), with a longer mean duration of readmissions (allo-HSCT 22.4 vs. 15.4 days, *p* < 0.001; SOT 11.6 vs. 7.5 days, *p* = 0.003). Comorbidity burden in transplantation patients was substantial, with several diagnoses being significantly more common among patients with CMV vs. non-CMV. One-year mortality did not differ significantly between patients with/without CMV.

**Conclusion:**

Burden of transplant recipients with CMV in terms of rehospitalizations and comorbidities is substantial, highlighting the need for improved CMV prevention and treatment.

**Supplementary Information:**

The online version contains supplementary material available at 10.1007/s15010-022-01847-2.

## Introduction

Cytomegalovirus (CMV), a beta herpes virus, may reside as a dormant virus in the majority of people, with estimated seroprevalences of almost 60% in Germany and the USA [1, 2] and an estimated global seroprevalence of 83% among the general population [3]. After initial infection, the virus remains persistent within the host, with complications that are most common in individuals with a suppressed immune system, such as recipients of solid organ transplantation (SOT) or allogeneic hematopoietic stem cell transplantation (allo-HSCT) [4, 5]. CMV is one of the most significant infectious pathogens in immunocompromised transplant recipients [5]. While CMV infection alone is defined as the detection of viral particles in the body fluid or tissue, patients with CMV disease show clinical symptoms or signs [6]. CMV disease consists of CMV syndrome and “end-organ disease”, which is characterized by the involvement of organs (e.g., pneumonia, gastrointestinal infections, hepatitis, or others) [6, 7]. Studies found that CMV infection occurred in around 22–34% of patients following HSCT, while 2–4% were diagnosed with CMV disease [8–10]. In SOT patients, rates of CMV infection generally depend on the type of organ transplant, serostatus of donor and recipient, and applied prevention strategies [11, 12]. Studies have reported CMV disease rates of 6–19% [13–16], while CMV infection rates vary between 16 and 26% [16, 17].

Without anti-CMV prophylaxis, CMV typically occurs during the first three months after transplantation [11, 18]. CMV infections result in high risk of graft-versus-host disease (GvHD) in HSCT patients, graft loss in SOT recipients, as well as other complications, morbidity, and mortality [19–21]. Thereby, CMV depicts a transplant-associated complication with a detrimental impact on the overall transplantation outcome [22]. On the other hand, complications like GvHD can increase the risk of CMV replication [23, 24]. Improvements in CMV management have been achieved in SOT patients via antiviral prophylaxis, applying valganciclovir and ganciclovir for 3 to 12 months post-transplantation [19]. Preemptive strategies were found to be similarly effective as prophylaxis in preventing graft loss and death among SOT patients [25]. Despite a significant reduction in the risk of CMV disease and mortality, prophylaxis after SOT has been linked to high rates of delayed-onset CMV after prophylaxis cessation [5, 26]. Long-term prophylaxis is, however, not feasible as it leads to hematologic toxicities such as neutropenia, thrombocytopenia, and anemia [5, 27]. Other antivirals used for the treatment of CMV, such as foscarnet and cidofovir, may lead to nephrotoxicity [19]. Additionally, the emergence of viral resistance can lead to failure of prophylaxis or breakthrough CMV infection. Until recently, preemptive treatment was the preferred approach in allo-HSCT patients due to the myelotoxic effects of prophylactic antivirals [6]. With the approval of letermovir in 2018, a viable prophylaxis option has been introduced for HSCT patients [26].

To date, data describing CMV frequency after allo-HSCT and SOT, including a characterization of respective patients, remains limited. This study aims to descriptively analyze the burden of transplantation recipients with subsequent CMV diagnoses using German claims data, with a specific focus on post-transplantation rehospitalizations and comorbidity burden.

## Methods

### Data source and sample selection

This retrospective study was based on an anonymized claims dataset covering the period from July 1, 2014, until June 30, 2019, provided by AOK PLUS, a regional German statutory health insurance fund insuring approximately 3.4 million people (4.7% of the statutorily insured German population). German claims data provide information on patients’ demographics (age, gender, date of death) and detailed reimbursement claims on outpatient care, inpatient care, pharmaceutical treatments, and other healthcare services covered by statutory health insurance.

The two key requirements for inclusion in the sample were continuous insurance throughout the entire study period from July 1, 2014, to June 30, 2019 (patients who died before the end of the study period were included) and a documented transplantation procedure between July 1, 2015, and June 30, 2018. Operation and procedure key (German: Operationen- und Prozedurenschlüssel, OPS) codes recorded during an inpatient stay were used to identify patients who underwent allo-HSCT (OPS: 8–863, 5-411.2–5.411.5, 8-805.2–8-805.5) or SOT (OPS: 5–555, 5–335, 5–375, 5–467.6, 5–504, 5–528.2). The date of admission for the transplantation-related hospital stay was defined as the index date, and patients were followed for 12 months post-index or until death, whichever came first.

### Post-transplantation CMV diagnoses

The presence of CMV infection or disease was defined via at least one documented inpatient diagnosis, or one confirmed outpatient diagnosis of CMV based on the International Classification of Diseases, Tenth Revision, German Modification (ICD-10-GM codes: B25.-, B27.1) during the 12 months follow-up period after the initial hospital admission. The frequency of CMV infections (identified via confirmed outpatient and inpatient diagnoses, ICD-10-GM codes: B25.-, B27.1) during the 12 months follow-up period was evaluated. A Kaplan–Meier analysis was conducted to depict the probability of CMV diagnosis after transplantation (failure function), with censorship of observations after 12 months or earlier, if the patient died within 12 months. The exact date of the CMV diagnosis was not available in the inpatient setting. For patients who received a CMV diagnosis during their initial hospitalization, the diagnosis date was therefore approximated as the midpoint of the initial hospital length of stay (LOS).

### Patient characteristics and index hospitalization

Baseline characteristics of included patients were descriptively analyzed for each transplantation group based on their respective index date or the 12 months pre-index period. Described variables included age, sex, comorbidities based on the Charlson Comorbidity Index [28] (CCI; described in Table S1), and the ten comorbidities not covered by the CCI, which were most frequently diagnosed within the entire transplantation sample, based on the first three ICD-10-GM code digits. Diagnoses generally present in allo-HSCT patients were not tracked in patients with the respective transplant (as not considered comorbidities). The index hospitalization was characterized in terms of LOS at the hospital, CMV diagnosis rate, and death rate during the index inpatient stay.

### Further post-transplantation outcomes

For the reporting of outcomes, patients were grouped according to transplantation type (allo-HSCT/SOT) and by CMV diagnosis status (CMV diagnosis observed during the 12 months post-index period: yes/no). The group of SOT recipients was comprised of patients receiving a variety of organs. In addition to results for the overall SOT group, the reports were supplemented by data separately analyzed for renal transplantations, which account for approximately 50% of SOTs in Germany [29], and non-renal SOT patients.

All-cause rehospitalizations after discharge from the initial hospital stay for transplantation were assessed in terms of rehospitalization rates. The number of rehospitalizations and total rehospitalization days in the 12 months post-index period were reported per patient-year (ppy), accounting for varying observable follow-up times due to death. In addition, the total number of hospitalization days ppy (including the index hospitalization) was reported. The duration of documented all-cause rehospitalizations was reported, as well as LOS of CMV-related rehospitalizations (CMV as main or primary diagnosis).

Comorbidities during follow-up were depicted using the CCI as well as the ten most frequently diagnosed comorbidities in the entire transplantation sample not covered by the CCI, based on the first three ICD-10-GM code digits. For this analysis, the index date, including the index hospitalization stay, was considered as part of the follow-up period. Furthermore, the proportion of patients with diagnoses of specific concomitant morbidities during follow-up was evaluated. Concomitant morbidities were defined by the presence of at least one inpatient diagnosis or confirmed outpatient diagnosis of renal impairment (ICD-10-GM: N14.-, N17.-, N18.-, N19.-, N28.9, N99.-) or bone marrow (BM) depression including neutropenia (ICD-10-GM: D70.-), thrombocytopenia (ICD-10-GM: D69.3, D69.4, D69.5, D69.6), and aplastic anemia (ICD-10-GM: D60.-, D61.-). As the need for a kidney transplant is inherently accompanied by a diagnosis of renal impairment, outcomes were calculated separately for renal and non-renal SOT, and renal impairment diagnoses were not shown for patients with a respective transplant. For the same reason, BM depression was not analyzed in allo-HSCT patients.

### Statistical analysis

For all categorical variables, the number and percentage of patients in each category were reported. Summary statistics, including mean, standard deviation (SD), and median, were applied for all continuous variables. Baseline characteristics and outcomes were compared between patients with/without CMV diagnosis using Fisher’s exact test for categorical variables and Mann–Whitney *U* test for continuous variables. Variables reported as rates ppy were compared using confidence intervals of the rate ratios based on estimated standard errors with 1000 bootstrap replications. *P* values < 0.05 were considered significant. Comparisons were not adjusted for the difference in the baseline patient characteristics. The analysis was performed using Microsoft Excel^®^ for Microsoft 365 MSO, and Stata software version 17.0 (StataCorp. 2021. Stata Statistical Software: Release 17. College Station, TX: StataCorp LLC).

## Results

### Study cohort

Approximately 2,22 million people were continuously insured for the entire study period between July 1, 2014, until June 30, 2019. Of these, 226 people received an allo-HSCT, and 250 underwent an SOT. Renal SOT was received by 128 patients, while 122 patients received a non-renal transplant (Supplementary Table S2). Almost half of the non-renal SOT patients received a liver transplant, 22% underwent lung transplantation, 24% received a heart or heart–lung transplant, and 7% were pancreas transplant recipients. None of the identified patients received transplantation of the small intestine.

### Post-transplantation CMV diagnoses

**Fig. 1:**
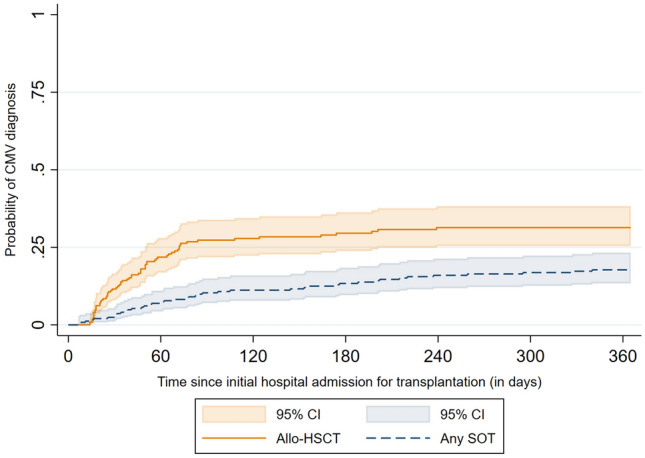
Kaplan–Meier failure curve of the estimated probability of receiving a CMV diagnosis after initial hospital admission for transplantation for allo-HSCT and SOT patients. Abbreviations: *Allo-HSCT* allogeneic hematopoietic stem cell transplantation, *CI* confidence interval, *CMV* cytomegalovirus, *SOT* solid organ transplantation

During the 12 months after hospital admission for transplantation, 66 allo-HSCT patients (29.2%) received a CMV diagnosis, while the rate of CMV diagnosis was 16.8% in the group of SOT patients. For the majority of allo-HSCT and non-renal SOT patients with CMV during follow-up, the diagnosis was documented during the initial hospital stay (53.0%/50.0% for allo-HSCT/non-renal SOT, respectively) or after discharge within the first three months after initial hospital admission (36.4%/18.2%; Supplementary Table S2). Among renal SOT patients with CMV, 10.0% received the diagnosis during the initial hospital stay, while another 40.0% were diagnosed within the following 3 months. Allo-HSCT patients were significantly more likely to receive a CMV diagnosis at any point in time during the first year after transplantation (HR 2.10, 95% CI 1.43–3.10, *p* < 0.001;Fig. 1). After 51 days (95% CI 38–72), 20% of allo-HSCT patients had received a CMV diagnosis. The rate of CMV diagnosis was not significantly different between patients with renal SOT and non-renal SOT (HR 1.27, 95% CI 0.69–2.33, *p* = 0.436; Supplementary Fig. S1).

Within one year after transplantation, 35.8% of patients who received a stem cell transplant died, while the death rate of SOT recipients was 10.8%. Death rates did not differ significantly between patients with and without CMV (allo-HSCT 37.9 vs. 35.0%, *p* = 0.761; SOT 11.9 vs. 10.6%, *p* = 0.787; Table 3).

### Patient characteristics

Patient baseline characteristics can be found in Table 1. The mean age at transplantation was 52.5 years for allo-HSCT patients and 53.0 years for SOT patients. The majority of transplant recipients were male, with 41.6%/36.4% of allo-HSCT/SOT patients being female, respectively. Comorbidity burden was generally high, with a mean CCI score of 4.6 in SOT patients and 4.2 in allo-HSCT patients. Baseline characteristics at index were not significantly different between patients with and without CMV diagnosis at follow-up, even if there was a trend towards a higher age and comorbidity status in the CMV groups.

**Table 1:** Main characteristics of patient samples, comorbidities most frequently diagnosed during the baseline period, and description of index hospitalization

	Allo-HSCT				SOT			
	All patients *n* = 226 Ø PYs = 0.77	CMV *n* = 66 Ø PYs = 0.79	No CMV *n* = 160 Ø PYs = 0.77	*p* value	All patients *n* = 250 Ø PYs = 0.92	CMV *n* = 42 Ø PYs = 0.94	No CMV *n* = 208 Ø PYs = 0.92	*p* value
Main characteristics								
Age in years, mean (SD)	52.5 (16.1)	54.1 (14.9)	51.9 (16.5)	0.320	53.0 (13.1)	54.6 (13.2)	53.7 (13.4)	0.430
Female patients, *n* (%)	94 (41.6)	30 (45.4)	64 (40.0)	0.462	91 (36.4)	14 (33.3)	77 (37.0)	0.727
CCI^a^, mean (SD)	4.2 (2.8)	4.3 (2.6)	4.2 (2.9)	0.667	4.6 (2.3)	5.0 (2.5)	4.5 (2.3)	0.242
Ten most frequently diagnosed comorbidities (including ICD-10-GM code), *n* (%)								
I10.- Essential (primary) hypertension	114 (50.4)	34 (51.5)	80 (50.0)	0.884	203 (81.2)	36 (85.7)	167 (80.3)	0.519
D69.- Purpura and other hemorrhagic conditions^b^	–	–	–	–	35 (14.0)	10 (23.8)	25 (12.0)	0.053
D70.- Agranulocytosis^b^	–	–	–	–	4 (1.6)	1 (2.4)	3 (1.4)	0.523
E78.- Disorders of lipoprotein metabolism and other lipidemias	54 (23.9)	15 (22.7)	39 (24.4)	0.865	109 (43.6)	22 (52.4)	87 (41.8)	0.234
M54.- Dorsalgia	97 (42.9)	35 (53.0)	62 (38.8)	0.055	62 (24.8)	11 (26.2)	51 (24.5)	0.846
D61.- Other aplastic anemias^b^	–	–	–	–	8 (3.2)	1 (2.4)	7 (3.4)	1.000
D64.- Other anemias^b^	–	–	–	–	70 (28.0)	14 (33.3)	56 (26.9)	0.452
E79.- Disorders of purine and pyrimidine metabolism	49 (21.7)	18 (27.3)	31 (19.4)	0.215	77 (30.8)	14 (33.3)	63 (30.3)	0.716
K29.- Gastritis and duodenitis	39 (17.3)	13 (19.7)	26 (16.3)	0.564	86 (34.4)	15 (35.7)	71 (34.1)	0.860
K76.- Other diseases of liver	50 (22.1)	16 (24.2)	34 (21.3)	0.371	70 (28.0)	13 (31.0)	57 (27.4)	0.707
Index hospitalization								
LOS, mean (SD)	42.8 (22.4)	45.1 (20.3)	41.0 (23.3)	0.173	45.9 (58.7)	43.9 (37.3)	46.3 (62.2)	0.207
CMV diagnosis, *n* (%)	35 (15.5)	35 (53.0)	NA	–	13 (5.2)	13 (31.0)	NA	–
Death, *n* (%)	24 (10.6)	4 (6.1)	20 (12.5)	0.234	18 (7.2)	2 (4.8)	16 (7.7)	0.746

*Allo-HSCT* allogeneic hematopoietic stem cell transplantation, *CMV* cytomegalovirus, *NA* not applicable, *PY* patient-year, *SD* standard deviation, *SOT* solid organ transplantation

^a^Charlson comorbidity index, not adjusted for age

^b^Diagnoses generally present in HSCT patients not shown for respective patient group

Co-diagnoses most frequently observed within the entire sample were essential (primary) hypertension, followed by hemorrhagic conditions, agranulocytosis, lipidemias, and dorsalgia. Hypertension was present in a majority of both allo-HSCT and SOT patients (50.4% and 81.2%, respectively). Other comorbidities frequently diagnosed in transplantation patients were disorders of lipoprotein metabolism and other lipidemias (allo-HSCT 23.9%, SOT 43.6%) and dorsalgia (allo-HSCT 42.9%, SOT 24.8%). At baseline, the proportion of patients experiencing certain comorbidities was not significantly different between those who received a CMV diagnosis during follow-up and those who did not, with borderline significance for hemorrhagic conditions in SOT patients (23.8 vs. 12.0%, *p* = 0.053) and dorsalgia in allo-HSCT recipients (53.0 vs. 38.8%, *p* = 0.055).

### Index hospitalization

Mean LOS at the index hospital was 42.8 days (SD 22.4) for allo-HSCT recipients and 45.9 days (SD 58.7) for SOT patients (Table 1). Hereby, LOS for non-renal SOT patients was 68.2 days (SD 76.6) on average, while recipients of renal transplants spent a mean of 24.6 days (SD 14.7) at the hospital (Supplementary Table S2). The difference was mainly driven by the long stays of heart/heart–lung patients who spent a mean of 148.7 days (SD 112.9) at the hospital for transplantation, while the mean LOS ranged from 38.0 to 46.0 days for lung, pancreas, and liver patients. Mortality rates during the initial hospital stay were 10.6% and 7.2% for allo-HSCT and SOT, respectively (Table 1). LOS and mortality rates during index hospitalization did not differ significantly between patients with/without CMV diagnosis during follow-up.

### Post-transplantation rehospitalizations

During the 12 months follow-up period after the index hospital admission, 182 allo-HSCT patients (80.5%) and 208 SOT patients (83.2%) were readmitted to the hospital (Table 2). Within the sample of allo-HSCT patients, the rehospitalization rate was significantly higher among patients with CMV during the follow-up period compared to those without a respective diagnosis (90.7 vs. 76.3%, *p* = 0.004). Among SOT recipients, only a numerical difference was observed (90.5 vs. 81.7%, *p* = 0.121).

**Table 2:** Rehospitalizations during the 12-month follow-up period after initial admission for transplantation

	Allo-HSCT				SOT			
	All patients *n* = 226 PYs = 174.9	CMV *n* = 66 PYs = 51.9	No CMV *n* = 160 PYs = 122.9	*p* value	All patients *n* = 250 PYs = 231.0	CMV *n* = 42 PYs = 39.5	No CMV *n* = 208 PYs = 191.5	*p* value
Patients with a rehospitalization, *n* (%)	182 (80.5)	60 (90.9)	122 (76.3)	0.004	208 (83.2)	38 (90.5)	170 (81.7)	0.121
Number of all-cause rehospitalizations, *n*	650	232	418	–	708	143	565	–
LOS (all-cause) in days, mean (SD) | median	17.9 (28.0) | 4.5	22.4 (33.0) | 8	15.4 (24.5) | 3	< 0.001	8.4 (16.3) | 3	11.6 (16.1) | 6	7.5 (16.2) | 3	0.003
Number of CMV-related rehospitalizations^b^, *n*	–	61	–	–	–	41	–	–
LOS (CMV-related)^b^ in days, mean (SD) | median	–	39.3 (42.1) | 23	–	–	–	23.0 (19.1) | 20	–	–
Number of all-cause rehospitalizations, ppy (95% CI)	3.7 (3.2–4.3)	4.5 (3.7–5.3)	3.4 (2.7–4.1)	0.050	3.1 (2.7–3.4)	3.6 (2.9–4.4)	3.0 (2.6–3.3)	0.116
Total all-cause rehospitalization days, ppy (95% CI)	62.4 (52.2–72.7)	93.3 (70.1–116.6)	49.4 (39.3–59.4)	0.001	24.3 (20.1–28.6)	42.0 (27.4–56.5)	20.7 (16.7–24.7)	0.005
Total all-cause hospitalization days^a^, ppy (95% CI)	117.8 (106.0–129.5)	150.6 (125.1–176.1)	103.9 (91.8–116.0)	0.001	73.9 (64.8–83.0)	88.6 (69.7–107.6)	70.9 (60.7–81.1)	0.101

*Allo-HSCT* allogeneic hematopoietic stem cell transplantation, *CI* confidence interval, *CMV* cytomegalovirus, *LOS* length of stay, *PY* patient year, *ppy* per patient-year, *SD* standard deviation, *SOT* solid organ transplantation

^a^Total hospitalization days during the 12 months post-index period including days spent at the hospital during the index admission for transplantation

^b^CMV-related defined as rehospitalizations with CMV as main or primary diagnosis

On average, allo-HSCT and SOT patients experienced 3.7 and 3.1 hospitalizations ppy, respectively. The number of all-cause readmissions ppy was numerically higher for patients with CMV during follow-up, with borderline significance in the allo-HSCT group (4.5 vs. 3.4, *p* = 0.050). Within the group of allo-HSCT recipients, the total time spent at the hospital for rehospitalization as well as the number of total hospitalization days (including the index admission) were significantly higher among patients with CMV (93.3 vs. 49.4 days, *p* = 0.001; 150.6 vs. 103.9 days, *p* = 0.001). SOT recipients with CMV during follow-up spent significantly more time at the hospital for readmissions (42.0 vs. 20.7 days, *p* = 0.005), while no statistical significance was observed for the number of total hospitalization days including the index admission (88.6 vs. 70.9 days, *p* = 0.101).

The mean duration of all-cause readmissions during the follow-up year was 17.9 days for allo-HSCT patients and 8.4 days for SOT recipients. These were, on average, significantly longer for patients with CMV compared to those without a CMV diagnosis in both allo-HSCT patients (22.4 vs. 15.4 days, *p* < 0.001) and SOT recipients (11.6 vs. 7.5 days, *p* = 0.003). Seventy-three patients (67.6%) who received a CMV diagnosis during the follow-up period were readmitted with CMV as the main or a primary diagnosis. The mean LOS for CMV-related readmissions was 39.3 days for allo-HSCT patients and 23.0 days for recipients of SOT (Table 2). Rehospitalization data by type of SOT (renal and non-renal) can be found in Supplementary Table S3.

### Post-transplantation morbidities

Co-diagnoses most frequently observed within the entire sample during the 12-month follow-up period can be found in Table 3. Similar to baseline, hypertension, hemorrhagic conditions, and agranulocytosis were among the five most frequently observed comorbidities. A substantial number of patients were diagnosed with ICD-10-GM code T86.- “Failure and rejection of transplanted organs and tissues” during follow-up. Notably, this code entails organ dysfunction, delayed integration of organs, and GvHD apart from true failure and rejection of transplants. Another condition frequently diagnosed during follow-up was”Disorders of fluid, electrolyte, and acid–base balance” (ICD-10-GM code: E87.-). The rates of these diagnoses were higher among patients with CMV compared to patients without a CMV diagnosis during follow-up. This was true for both allo-HSCT recipients (T86.-: 69.7 vs. 45.0%, *p* = 0.001; E87.-: 75.8 vs. 53.1%, *p* = 0.002) and SOT patients (T86.-: 85.7 vs. 64.9%, *p* = 0.010; E87.-: 81.0 vs. 55.3%, *p* = 0.002). Within the group of allo-HSCT recipients, CMV patients also showed higher rates of disorders of the urinary system (51.5 vs. 26.3%, *p* < 0.001). The rates of other comorbidities as well as the CCI during follow-up were not significantly different between patients with and without CMV.

**Table 3:** CCI, 10 most frequent comorbidities based on ICD-10-GM code, and death rate during the 12 months follow-up

	Allo-HSCT				SOT			
	All patients *n* = 226	CMV *n* = 66	No CMV *n* = 160	*p* value	All patients *n* = 250	CMV *n* = 42	No CMV *n* = 208	*p* value
CCI^a^, mean (SD)	5.3 (2.9)	5.5 (2.6)	5.2 (3.1)	0.262	5.8 (3.1)	6.0 (3.0)	5.7 (3.1)	0.474
I10.- Essential (primary) hypertension	123 (54.4)	40 (60.6)	83 (51.9)	0.244	206 (82.4)	36 (85.7)	170 (81.7)	0.660
T86.- Failure and rejection of transplanted organs and tissues^b^	118 (52.2)	46 (69.7)	72 (45.0)	0.001	171 (68.4)	36 (85.7)	135 (64.9)	0.010
E87.- Other disorders of fluid, electrolyte and acid–base balance	135 (59.7)	50 (75.8)	85 (53.1)	0.002	149 (59.6)	34 (81.0)	115 (55.3)	0.002
D69.- Purpura and other hemorrhagic conditions^c^	–	–	–	–	67 (26.8)	15 (35.7)	52 (25.0)	0.181
D70.- Agranulocytosis^c^	–	–	–	–	29 (11.6)	8 (19.0)	21 (10.1)	0.113
D61.- Other aplastic anemias^c^	–	–	–	–	10 (4.0)	4 (9.5)	6 (2.9)	0.067
E78.- Disorders of lipoprotein metabolism and other lipidemias	40 (17.7)	9 (13.6)	31 (19.4)	0.344	136 (54.4)	26 (61.9)	110 (52.9)	0.312
D68.- Other coagulation defects	62 (27.4)	23 (34.8)	39 (24.4)	0.076	114 (45.6)	19 (45.2)	95 (45.7)	0.548
N39.- Other disorders of urinary system	76 (33.6)	34 (51.5)	42 (26.3)	< 0.001	97 (38.8)	17 (40.5)	80 (38.5)	0.863
E83.- Disorders of mineral metabolism	52 (23.0)	21 (31.8)	31 (19.4)	0.056	91 (36.4)	16 (38.1)	75 (36.1)	0.861
Death, *n* (%)	81 (35.8)	25 (37.9)	56 (35.0)	0.761	27 (10.8)	5 (11.9)	22 (10.6)	0.787

*Allo-HSCT* allogeneic hematopoietic stem cell transplantation, *CCI* Charlson Comorbidity Index, *PY* patient-year, *SD* standard deviation, *SOT* solid organ transplantation

^a^Charlson comorbidity index, not adjusted for age

^b^ICD-10-GM code includes documentation of graft-versus-host disease, transplant dysfunction, delayed integration of transplant, as well as transplant failure and rejection

^**c**^Diagnoses generally present in HSCT patients not shown for respective patient group

One-year mortality was 35.8% among allo-HSCT patients and 10.8% among SOT patients (Table 3). Hereby, mortality rates did not differ significantly between patients with CMV vs. those without CMV (allo-HSCT 37.9 vs. 35.0%, *p* = 0.761; SOT 11.9 vs. 10.6%, *p* = 0.787). Comorbidities and death rates during follow-up by type of SOT (renal and non-renal) can be found in Supplementary Table S4.

The rates of specific transplantation-related concomitant morbidities are depicted in Table 4. Approximately half of the allo-HSCT recipients (50.9%) and more than three-quarters of non-renal SOT patients (76.2%) received a diagnosis of renal impairment during follow-up. BM depression was diagnosed in 18.8% of renal SOT patients, where neutropenia was the most common diagnosis (10.2% of all renal SOT). More than half of the non-renal SOT patients (51.6%) were diagnosed with BM depression, with the most frequently diagnosed morbidity being thrombocytopenia (44.2% of all non-renal SOT), followed by neutropenia (13.1%). Significant differences between patients with and without CMV during the follow-up were found for renal impairment within the allo-HSCT group (66.7 vs. 44.4%, *p* = 0.003) and for neutropenia among renal SOT patients (25.0 vs. 7.4%, *p* = 0.032).

**Table 4:** Transplantation-related concomitant morbidities diagnosed among transplantation patients during the 12-month follow-up period

	Allo-HSCT				Renal SOT				Non-renal SOT			
	All patients *n* = 226	CMV *n* = 66	No CMV *n* = 160	*p* value	All patients *n* = 128	CMV *n* = 20	No CMV *n* = 108	*p* value	All patients *n* = 122	CMV *n* = 22	No CMV *n* = 100	*p* value
Renal impairment^a^	115 (50.9)	44 (66.7)	71 (44.4)	0.003	–	–	–	–	93 (76.2)	18 (81.8)	75 (75.0)	0.593
BM depression^b^	–	–	–	–	24 (18.8)	7 (35.0)	17 (15.7)	0.060	63 (51.6)	13 (59.1)	50 (50.0)	0.487
Neutropenia	–	–	–	–	13 (10.2)	5 (25.0)	8 (7.4)	0.032	16 (13.1)	3 (13.6)	13 (13.0)	1.000
Thrombocytopenia	–	–	–	–	11 (8.6)	2 (10.0)	9 (8.3)	0.682	54 (44.3)	11 (50.0)	43 (43.0)	0.638
Aplastic anemia	–	–	–	–	3 (2.3)	1 (5.0)	2 (1.9)	0.402	7 (5.7)	3 (13.6)	4 (4.0)	0.110

Data are *n* (%)

*Allo-HSCT* allogeneic hematopoietic stem cell transplantation, *BM* bone marrow, *CMV* cytomegalovirus, *SOT* solid organ transplantation

^a^Diagnoses generally present in renal SOT patients not shown for respective patient group

^b^Diagnoses generally present in HSCT patients not shown for respective patient group

## Discussion

This study investigated German claims data regarding the burden of patients with post-transplantation CMV. Within this study, 226 allo-HSCT recipients and 250 patients who received SOT within a time period of 3 years were identified. Almost one third of allo-HSCT patients and approximately 17% of SOT recipients were diagnosed with CMV during the 12 months after admission for transplantation. The results indicate that allo-HSCT patients who receive a CMV diagnosis after transplantation experience a significantly higher burden in terms of all-cause rehospitalizations compared to those without CMV diagnosis, while number and duration of rehospitalizations of SOT patients was found to be numerically higher.

Generally, not much evidence has been published regarding the burden of rehospitalizations faced by transplantation patients with CMV. However, three database studies conducted in the US and France that investigated outcomes similar to the ones reported in this publication were identified. A retrospective US database study by Schelfhout et al. [10] observed more than 1800 allo-HSCT patients over a 12 months period, comparing those with and those without a CMV diagnosis during follow-up. In our study, the rehospitalization rate was higher among allo-HSCT patients with CMV vs. those without (90.9 vs. 76.3%, *p* = 0.004). In line with our findings, Schelfhout et al. reported that allo-HSCT patients with CMV were significantly more likely to have an inpatient readmission during a 12-month follow-up period (78.9 vs. 57.8%, *p* < 0.001). While we found only borderline significance for the number of rehospitalizations ppy (4.5 vs. 3.4, *p* = 0.050), this study reported a significantly higher mean number of admissions (3.3 vs. 2.3, *p* < 0.001) within 12 months after the transplantation among CMV patients. In contrast to our data, the average duration of readmissions in this study was only numerically higher for patients with CMV (23.1 vs. 21.9 days, *p* = 0.191; our study: 22.4 vs. 15.4 days, *p* < 0.001). Schelfhout et al. included the index hospitalization in their calculations of number and duration of hospital admissions. This, and differences in the healthcare systems, may partly explain the differing findings. Another retrospective database study by Schelfhout et al. [30] observed allo-HSCT patients over a period of 100 post-transplantation days. While we found the average length of CMV-related admissions to be 39.3 days during follow-up, this study reported that CMV-related admissions lasted only 24.4 days on average during the first 100 post-transplantation days. The rates of CMV during the initial hospital admission were only 3.9% in this study, compared to 15.5% in our analysis. This may, in part, be explained by the longer initial hospital stay of allo-HSCT patients in our study (42.8 vs. 27.5 days in Schelfhout et al. [30]). Finally, Hakimi et al. [31] used a French database to identify SOT recipients who developed CMV disease in an inpatient setting and matched these to SOT patients without CMV disease. This study found that the number of hospital readmissions as well as the number of cumulative inpatient days was significantly higher among patients who developed CMV in an inpatient setting compared to those without CMV, irrespective of the timing of CMV onset.

In our study, 1-year mortality did not differ significantly between CMV and non-CMV patients (allo-HSCT 37.9 vs. 35.0%; SOT: 11.9 vs. 10.6%). In contrast to our study, much of the published literature reports an association of CMV infection and/or disease with decreased survival for both allo-HSCT [32, 33] and SOT patients (including kidney [34, 35] and liver recipients [36, 37]). Several studies reported that donor and/or recipient CMV serology alone was associated with a higher risk of mortality among HSCT [38, 39] and SOT patients [31, 40, 41]. On the other hand, Mabilangan et al. [42, 43] found no association between donor/recipient CMV serology and 10 years mortality among heart or lung transplant recipients. One potential explanation for the similar survival rates of CMV and non-CMV patients in our study may be the limited follow-up time of 1 year, which is comparatively shorter than other analyses of survival which reported outcomes based on extended follow-up periods of 5 years or longer [34–36, 39, 41]. Nonetheless, CMV serology, infection, and disease have all been correlated with increased mortality rates, measured from 6 months to 2 years post-transplantation [32, 33, 37, 38, 40]. Finally, the changing CMV treatment landscape may, in part, explain our results. Several studies suggesting an association of CMV with short-term survival were partly conducted prior to the introduction of valganciclovir [37, 40], which has been used as (prophylactic) treatment in SOT patients and is associated with reduced risk of long-term mortality [41–44]. Similarly, the more recently approved agent letermovir can now be applied as prophylaxis in allo-HSCT patients and may reduce mortality by preventing or delaying CMV infection [45, 46].

The second most common co-diagnosis among transplantation patients during follow-up was ”Failure and rejection of transplanted organs or tissues”, encompassing complications directly related to the transplantation. Based on published literature, graft loss or rejection commonly occur in 10–40% of kidney and pancreas transplantations, 20–50% of liver transplantations, and ≥ 50% of heart and lung transplantations [47, 48]. The ICD-10-GM code T86.- was claimed for 68.4% of SOT recipients in our study. Importantly, subcategories of the ICD-10-GM code T86.- include organ dysfunction and delayed integration of organs, which may account for a large proportion of observed diagnoses. Diagnoses of this class were significantly more common among SOT patients with CMV (85.7 vs. 64.9%). While based on these results, it is not possible to conclude whether CMV infection occurred before or after transplant failure/rejection/dysfunction, a study conducted in SOT patients by Hakimi et al. [31] suggests a causal relationship between CMV disease and decreased graft rejection-free and graft failure-free survival, irrespective of the timing of CMV onset. Based on published literature, graft failure commonly occurs in less than 6% of allo-HSCT patients [49]. In our analysis, transplant failure was coded for < 5% of patients overall and < 10% of patients diagnosed with T86.- while the remaining patients diagnosed with this code experienced various grades of GvHD. A significantly higher proportion of allo-HSCT patients with CMV was diagnosed with T86.- compared to those without CMV (69.7 vs. 45.0%), suggesting a potential association between CMV and GvHD. While in our analysis it was not possible to establish a causal relationship between CMV and GvHD, Cantoni et al. [23] suggest a bi-directional relationship in which GvHD and its therapy increase risk of CMV infection while the risk of developing GvHD is higher during CMV replication.

In our analysis, other disorders of fluid, electrolyte, and acid–base balance (ICD-10-GM: E87.-) constituted the third most frequent class of diagnoses given to patients during the year after the transplantation. Fluid and electrolyte problems are common complications following both allo-HSCT and SOT [50–52]. We found that while slightly more than half of the patients without CMV during follow-up received an E87.- diagnosis (allo-HSCT 53%; SOT 55%), three-quarters of allo-HSCT patients and 81% of SOT patients with CMV were diagnosed with a fluid/electrolyte/acid–base balance disorder, suggesting that CMV may play a role in promoting this type of post-transplant complication as well as known treatment-limiting toxicities of foscarnet and cidofovir which are used to manage CMV.

Due to the myelotoxic and/or nephrotoxic effects of available antiviral medications, renal impairment and BM depression preclude the intake of certain antivirals. Since patients in need of a kidney transplant inherently suffer from renal impairment, the applicability of foscarnet and cidofovir in these patients is limited. Our analysis showed that half of the included allo-HSCT patients and three-quarters of non-renal SOT patients were diagnosed with renal impairment, and BM depression was diagnosed in half of the included recipients of non-renal SOT. Within the group of allo-HSCT patients, the rate of renal impairment was significantly higher among patients with CMV (67 vs. 44%), while neutropenia was diagnosed in a significantly higher proportion of patients with CMV among renal SOT recipients (25 vs. 7%). As antiviral medication was not observable in the inpatient setting, it remains unclear how many patients suffered renal impairment and BM depression as an effect of antiviral medication or due to other causes. In any case, our data support the need for antivirals without myelotoxic and/or hepatotoxic/nephrotoxic properties. The recently approved antiviral agent letermovir has successfully been applied in clinical practice as CMV prophylaxis in allo-HSCT patients and is associated with less myelotoxicity than other CMV medications, potentially circumventing the common problem of antiviral resistance of CMV [46, 53–55]. Meanwhile, maribavir constitutes a potential antiviral agent for therapeutic treatment of CMV, which has shown numerically higher performance to valganciclovir in treating CMV infection without associated myelo- or nephrotoxicity [55–58].

### Strengths and limitations

To the knowledge of the authors, this is the first analysis of German claims data investigating the burden of patients with post-transplantation CMV. The main strength of our study is the use of a large, generalizable, representative claims dataset with over 3.4 million statutorily insured people in Germany. The dataset covers over 50% of inhabitants of the German states Saxony and Thuringia. In comparison to other real-world observational studies, this dataset was not affected by any site or patient selection bias.

Within our analyses, we confirmed rates of CMV after allo-HSCT and SOT identified in previous studies with other study designs, while additionally examining the timing of CMV onset. We furthermore found comparable results in Germany in terms of rehospitalization burden as were described in similar studies in France and the USA.

This study was designed as a descriptive analysis of characteristics and rehospitalization data associated with CMV; thus, no specific a-priori hypotheses were tested. Nonetheless, main characteristics observable in claims data including age, sex, and comorbidities at baseline were balanced between patient samples with/without CMV diagnosis, suggesting that CMV- and therapy-associated effects are the major cause for the observed morbidity differences. The evidence of longer hospital stays and more frequent readmission rates confirms an increased and clinically as well as health-economically relevant morbidity of transplant patients with CMV diagnosis, which was so far primarily shown on a single-center basis. Furthermore, our study illustrates the need for antivirals without myelotoxic and/or hepatotoxic properties, as many patients with CMV still suffer from these toxicities.

Several limitations to the study should be acknowledged. First and foremost, several limitations arose due to the limited availability of information in claims datasets. It was not possible to clearly distinguish between CMV infection and disease based on ICD-10-GM codes, and therefore, patients in our sample could only be defined by the presence of a CMV diagnosis. However, as CMV diseases are rare events relative to CMV infections, at least in the HSCT setting, we consider the lack of discriminatory power of our source data in this regard to be of little clinical relevance. Although some codes explicitly detail the involvement of an organ, the vast majority (84%) of patients in our sample received a diagnosis of miscellaneous or unspecified CMV disease (ICD-10-GM: B25.88, B25.9), and it was not clear whether patients who received these diagnoses suffered from an infection or disease. In addition, most antiviral agents could not be observed in the inpatient setting. As transplantation patients spend a considerable amount of time at the hospital, information on outpatient prescriptions for antivirals would not paint a comprehensive picture of patients’ treatment journeys. Therefore, antiviral treatment could not be analyzed in this study. This hampered our ability to evaluate the effect of antiviral prophylaxis and treatment on CMV onset and common toxicities. Notably, the treatment landscape of CMV prophylaxis in allo-HSCT patients has changed during recent years following the introduction of letermovir in 2018, with reduced CMV reactivation rates post-letermovir approval [26]. As our sample includes patients with a transplantation between July, 2015 and June, 2018, the majority of our patients received transplants during the pre-letermovir era. None of the patients included in our study was observed to receive letermovir in an inpatient (OPS 6-00b.c, 6-00b.d) or outpatient setting (ATC J05AX18) during the follow-up period. CMV and toxicity rates among allo-HSCT patients post-letermovir approval may therefore differ from results reported in our study.

Second, our dataset was provided by a regional sickness fund, covering the statutorily insured population in the German states Thuringia and Saxony, not covering privately insured people. Consequently, results may not be representative of the entire German population of transplant recipients. Nonetheless, no large differences in healthcare are expected between German federal states as the German healthcare system is considered rather homogenous, and therefore, it is not expected that findings would differ largely from other parts of Germany.

Finally, due to the limited number of transplant patients within our sample, a matching procedure of patient cohorts with and without CMV was not conducted; therefore, analyses were not adjusted for baseline characteristics. Although very few statistically significant differences were found between the comparison groups in terms of age, sex, and comorbidities at baseline, the conducted comparisons may suffer from confounding. Furthermore, adjustment for multiple testing has not been performed. For these reasons, individual comparisons must be interpreted with care. Nonetheless, the long average duration of CMV-related rehospitalizations (39.3 days for allo-HSCT and 23.0 days for SOT recipients) supports the overall picture of CMV being related to a higher rehospitalization burden.

## Conclusion

In our analysis, patients with CMV during the 12 months follow-up period spent significantly more time at the hospital for readmissions and experienced a numerically higher number of rehospitalizations compared to those without CMV, among both allo-HSCT and SOT recipients. Additionally, the hospitalization rate was significantly higher for patients with CMV among allo-HSCT patients. Our study illustrates the high comorbidity burden of transplant recipients, with significantly higher rates of fluid/electrolyte disorders among patients with CMV than among those without CMV. Ultimately, GvHD, renal impairment, and disorders of the urinary system were more commonly observed among allo-HSCT patients with CMV, while neutropenia was more frequently diagnosed among renal SOT patients with CMV. One-year mortality did not differ between patients with distinct CMV statuses during follow-up, which may be explained by the short follow-up time and emerging prophylaxis options made available to patients during recent years. Nevertheless, our analysis highlights the need for improved CMV prevention and treatment. The introduction of letermovir as CMV prophylaxis has dramatically shaped the CMV treatment landscape in recent years. Coupled with the recent approval of maribavir in the US in November 2021 and potential subsequent approval in Europe, a further analysis of CMV rates, rehospitalization, and treatment-limiting toxicities is warranted, to account for the evolving real-world use of both agents.

## Supplementary Information

Below is the link to the electronic supplementary material.

Supplementary file1 (DOCX 168 KB)
