# Supplementary material for: Cytomegalovirus infection and rehospitalization rates after allogeneic hematopoietic stem cell and solid organ transplantation: a retrospective cohort study using German claims data
Source: Infection. 2022 May 28;50(6):1543–55. doi: 10.1007/s15010-022-01847-2 (PMC9705421; doi:10.1007/s15010-022-01847-2)
Supplement: Supplementary file 1 — Supplementary file1 (DOCX 168 KB) [file 15010_2022_1847_MOESM1_ESM.docx]

**Cytomegalovirus infection and rehospitalization rates after allogeneic hematopoietic stem cell and solid organ transplantation: A retrospective cohort study using German claims data**

**Supplementary Material**

Daniel Teschner^1,2^, Jana Knop^3^, Christian Piehl^3^, Sophia Junker^4^, Oliver Witzke^5^

^1^ University Hospital Würzburg, Department of Internal Medicine II, Würzburg, Germany

^2^ University Medical Centre of the Johannes Gutenberg University Mainz, Department of Hematology, Medical Oncology, and Pneumology, Mainz, Germany

^3^ Takeda Pharma Vertrieb GmbH & Co. KG, Berlin, Germany

^4^ Ingress-Health HWM GmbH, a wholly owned subsidiary of Cytel Inc., Berlin, Germany

^5^ Department of Infectious Diseases, West German Centre of Infectious Diseases, University Hospital Essen, Essen, Germany; University of Duisburg-Essen

**Supplemental Table S1:** Charlson Comorbidity Score (CCI) and components

| **Comorbidity** | **ICD-10 code** | **Charlson score** |
| --- | --- | --- |
| Coronary artery disease | I20.-, I21.-, I22.-, I23.-, I24.-, I25.- | 1 |
| Congestive heart failure | I11.-, I50.- | 1 |
| Peripheral vascular disease | I73.-, I74.-, I77.- | 1 |
| Cerebrovascular disease | G45.-, G46.-, I6.- | 1 |
| Dementia | F00.-, F01.-, F02.-, F03.-, G30.- | 1 |
| Chronic pulmonary disease | J4.-, J6.- w/o J67.-, J68.-, J69.- | 1 |
| Connective tissue disorder | M05.-, M06.-, M07.-, M08.-, M3.- | 1 |
| Peptic ulcer disease | K25.-, K26.-, K27.-, K28.- | 1 |
| Mild liver disease | B18.-, K70.-, K73.-, K75.- | 1 |
| Diabetes mellitus without complications | E109.-, E119.-, E129.-, E139.-, E149.- | 1 |
| Hemiplegia | G81.-, G82.- | 2 |
| Moderate or severe renal disease | N17.-, N18.-, N19.- | 2 |
| Diabetes mellitus with end-organ damage | E10.-, E11.-, E12.-, E13.-, E14.- | 2 |
| Tumor without metastases, leukemia, lymphoma, multiple myeloma | C% w/o (77-80) | 2 |
| Moderate or severe liver disease | K72.-, K74.-, I85.- | 3 |
| Metastatic solid tumor | C77.-, C78.-, C79.-, C80.- | 6 |
| AIDS | B20.-, B21.-, B22.-, B23.-, B24.- | 6 |

**Supplemental Table S2**: Main characteristics of patient samples, comorbidities most frequently diagnosed during the baseline period, and description of index hospitalization of SOT patients, split by transplant type (renal/non-renal)

|  | Renal SOT |  |  | Non-renal SOT |  |  |
| --- | --- | --- | --- | --- | --- | --- |
|  | **All patients** n = 128  Ø PYs = 0.97 | **CMV** n = 20  Ø PYs = 0.99 | **No CMV** n = 108  Ø PYs = 0.96 | **All patients** n = 122  Ø PYs = 0.88 | **CMV** n = 22  Ø PYs = 0.90 | **No CMV** n = 100  Ø PYs = 0.88 |
| Age in years, mean (SD) | 54.0 (12.9) | 57.4 (12.1) | 53.4 (13.1) | 52.0 (13.7) | 52.1 (13.9) | 51.9 (13.8) |
| Female patients, n (%) | 48 (37.5) | 7 (35.0) | 41 (38.0) | 43 (35.2) | 7 (31.8) | 36 (36.0) |
| CCI^a^, mean (SD) | 4.2 (1.9) | 4.7 (2.0) | 4.1 (1.9) | 5.0 (2.6) | 5.2 (2.8) | 5.0 (2.6) |
| I10.- Essential (primary) hypertension | 117 (91.4) | 19 (95.0) | 98 (90.7) | 86 (70.5) | 17 (77.3) | 69 (69.0) |
| D69.- Purpura and other hemorrhagic conditions | 7 (5.5) | 2 (10.0) | 5 (4.6) | 28 (23.0) | 8 (36.4) | 20 (20.0) |
| D70.- Agranulocytosis | 0 (0.0) | 0 (0.0) | 0 (0.0) | 4 (3.3) | 1 (4.5) | 3 (3.0) |
| E78.- Disorders of lipoprotein metabolism and other lipidaemias | 63 (49.2) | 12 (60.0) | 51 (47.2) | 46 (37.7) | 10 (45.5) | 36 (36.0) |
| M54.- Dorsalgia | 32 (25.0) | 6 (30.0) | 26 (24.1) | 30 (24.6) | 5 (22.7) | 25 (25.0) |
| D61.- Other aplastic anemias | 3 (2.3) | 1 (5.0) | 2 (1.9) | 5 (4.1) | 0 (0.0) | 5 (5.0) |
| D64.- Other anemias | 46 (35.9) | 9 (45.0) | 37 (34.3) | 24 (19.7) | 5 (22.7) | 19 (19.0) |
| E79.- Disorders of purine and pyrimidine metabolism | 51 (39.8) | 7 (35.0) | 44 (40.7) | 26 (21.3) | 7 (31.8) | 19 (19.0) |
| K29.- Gastritis and duodenitis | 32 (25.0) | 5 (25.0) | 27 (25.0) | 54 (44.3) | 10 (45.5) | 44 (44.0) |
| K76.- Other diseases of liver | 17 (13.3) | 4 (20.0) | 13 (12.0) | 53 (43.4) | 9 (40.9) | 44 (44.0) |
| LOS, mean (SD) | 24.6 (14.7) | 29.2 (18.1) | 23.7 (14.0) | 68.2 (76.6) | 57.3 (44.9) | 70.7 (81.9) |
| CMV diagnosis, n (%) | 2 (1.6) | 2 (10.0) | 0 (0.0) | 11 (9.0) | 11 (50.0) | 0 (0.0) |
| Death, n (%) | 3 (2.3) | 0 (0.0) | 3 (2.8) | 15 (12.3) | 2 (9.1) | 13 (13.0) |
| Lung transplantation, n (%) | - | - | - | 27 (22.1) | 4 (3.3) | 23 (18.9) |
| Heart-lung transplantation, n (%) | - | - | - | 29 (23.8) | 4 (4.4) | 25 (20.5) |
| Liver transplantation, n (%) | - | - | - | 58 (47.5) | 11 (9.0) | 47 (38.5) |
| Pancreas transplantation, n (%) | - | - | - | 8 (6.6) | 3 (2.5) | 5 (4.1) |

^a^ Charlson comorbidity index, not adjusted for age.

CMV, cytomegalovirus; SD, standard deviation; PY, patient-year; SOT, solid organ transplantation

**Supplemental Table S3**: Rehospitalizations during the 12-month follow-up period after initial admission for transplantation in SOT patients, split by transplant type (renal/non-renal)

|  | Renal SOT |  |  | Non-renal SOT |  |  |
| --- | --- | --- | --- | --- | --- | --- |
|  | **All patients** n = 128 PYs = 123.7 | **CMV** n = 20 PYs = 19.8 | **No CMV** n = 108 PYs = 103.9 | **All patients** n = 122 PYs = 107.3 | **CMV** n = 22 PYs = 19.7 | **No CMV** n = 100 PYs = 87.5 |
| Patients with a rehospitalization, n (%) | 116 (90.6) | 19 (95.0) | 97 (89.8) | 92 (75.4) | 19 (86.4) | 73 (73.0) |
| Number of all-cause rehospitalizations, ppy (95% CI) | 3.2 (2.8–3.5) | 3.7 (2.8–4.7) | 3.0 (2.7–3.4) | 3.0 (2.8–3.5) | 3.5 (2.3–4.7) | 2.8 (2.2–3.5) |
| Total all-cause rehospitalization days, ppy (95% CI) | 24.1 (19.4–28.8) | 44.0 (27.3–60.7) | 20.3 (16.1–24.5) | 24.6 (17.1–32.0) | 39.9 (15.7–64.2) | 21.1 (13.9–28.3) |
| Total all-cause hospitalization days^a^, ppy (95% CI) | 49.5 (43.5–55.6) | 73.5 (53.6–93.4) | 45.0 (39.2–50.7) | 102.1 (85.0–119.2) | 103.8 (71.9–135.7) | 101.7 (82.0–121.5) |
| Number of all-cause rehospitalizations, n | 390 | 74 | 316 | 318 | 69 | 249 |
| LOS (all-cause) in days, mean (SD) \| median | 7.7 (9.8) \| 4 | 11.8 (13.0) \| 7 | 6.8 (8.6) \| 3 | 9.1 (21.7) \| 3 | 11.4 (19.0) \| 4 | 8.5 (22.4) \| 2 |
| Number of CMV-related rehospitalizations^b^, n | - | 21 | - | - | 20 | - |
| LOS (CMV-related)^b^ in days, mean (SD) \| median | - | 22.4 (15.0) \| 24 | - | - | 23.7 (23.0) \| 17 | - |

**^a^** Total hospitalization days during the 12-month post-index period including days spent at the hospital during the index admission for transplantation; **^b^** CMV-related defined as rehospitalizations with CMV as main or primary diagnosis

CI, confidence interval; CMV, cytomegalovirus; LOS, length of stay; PY, patient year; ppy, per patient-year; SD, standard deviation; SOT, solid organ transplantation

**Supplemental Table S4:** Comorbidity burden (CCI and 10 most frequent diagnoses including ICD-10-GM code) and death rate during the 12-month follow-up period among SOT patients, split by transplant type (renal/non-renal)

|  | Renal SOT |  |  | Non-renal SOT |  |  |
| --- | --- | --- | --- | --- | --- | --- |
|  | **All patients** n = 128 PYs = 123.7 | **CMV** n = 20 PYs = 19.8 | **No CMV** n = 108 PYs = 103.9 | **All patients** n = 122 PYs = 107.3 | **CMV** n = 22 PYs = 19.7 | **No CMV** n = 100 PYs = 87.5 |
| CCI^a^, mean (SD) | 4.9 (2.7) | 5.3 (2.1) | 4.8 (2.8) | 6.7 (3.2) | 6.7 (3.6) | 6.7 (3.2) |
| I10.- Essential (primary) hypertension | 120 (93.8) | 18 (90.0) | 102 (94.4) | 86 (70.5) | 18 (81.8) | 68 (68.0) |
| T86.- Failure and rejection of transplanted organs and tissues | 100 (78.1) | 19 (95.0) | 81 (75.0) | 71 (58.2) | 17 (77.3) | 54 (54.0) |
| E87.- Other disorders of fluid, electrolyte and acid-base balance | 69 (53.9) | 14 (70.0) | 55 (50.9) | 80 (65.6) | 20 (90.9) | 60 (60.0) |
| D69.- Purpura and other hemorrhagic conditions | 12 (9.4) | 3 (15.0) | 9 (8.3) | 55 (45.1) | 12 (54.5) | 43 (43.0) |
| D70.- Agranulocytosis | 13 (10.2) | 5 (25.0) | 8 (7.4) | 16 (13.1) | 3 (13.6) | 13 (13.0) |
| D61.- Other aplastic anemias | 3 (2.3) | 1 (5.0) | 2 (1.9) | 7 (5.7) | 3 (13.6) | 4 (4.0) |
| E78.- Disorders of lipoprotein metabolism and other lipidemias | 85 (66.4) | 16 (80.0) | 69 (63.9) | 51 (41.8) | 10 (45.5) | 41 (41.0) |
| D68.- Other coagulation defects | 23 (18.0) | 4 (20.0) | 19 (17.6) | 91 (74.6) | 15 (68.2) | 76 (76.0) |
| N39.- Other disorders of urinary system | 58 (45.3) | 10 (50.0) | 48 (44.4) | 39 (32.0) | 7 (31.8) | 32 (32.0) |
| E83.- Disorders of mineral metabolism | 57 (44.5) | 9 (45.0) | 48 (44.4) | 34 (27.9) | 7 (31.8) | 27 (27.0) |
| Death, n (%) | 6 (4.7) | 1 (5.0) | 5 (4.6) | 21 (17.2) | 4 (18.2) | 17 (17.0) |

^a^ Charlson comorbidity index, not adjusted for age.

CCI, Charlson Comorbidity Index; PY, patient-year; SD, standard deviation; SOT, solid organ transplantation

**Supplemental Fig. S1:** Kaplan-Meier failure curve of the estimated probability of receiving a CMV diagnosis after initial hospital admission for transplantation for allo-HSCT, renal SOT recipients, and non-renal SOT patients


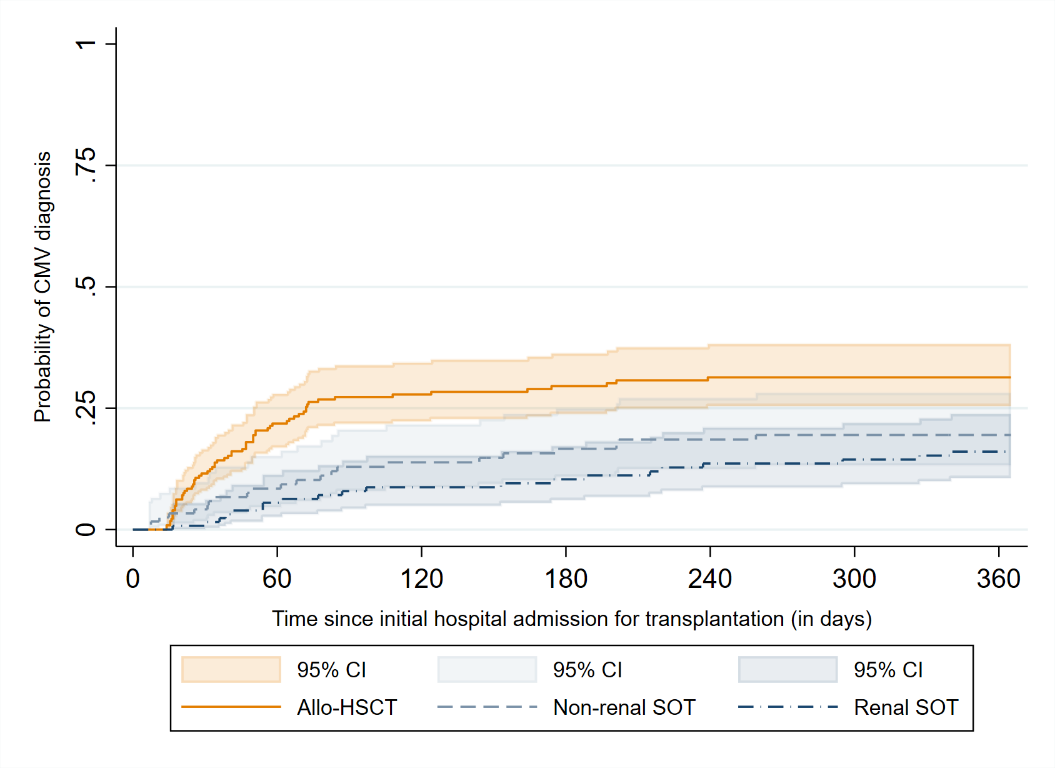


Allo-HSCT, allogeneic hematopoietic stem cell transplantation; CI, confidence interval; CMV, cytomegalovirus; SOT, solid organ transplantation
